# Assessing the Efficacy of RADPAD Protection Drape in Reducing Radiation Exposure to Operators in the Cardiac Catheterization Laboratory: A Systematic Review and Meta-Analysis

**DOI:** 10.7759/cureus.59215

**Published:** 2024-04-28

**Authors:** Abdul Rasheed Bahar, Resha Khanal, Mohammad Hamza, Rohit K Goru, Aimen Shafiq, Mobeen Z Haider, Salman Abdul Basit, Yasemin Bahar, Ahmed Muaaz Umer, Yasar Sattar, M. Chadi Alraies

**Affiliations:** 1 Internal Medicine, Wayne State University Detroit Medical Center, Detroit, USA; 2 Internal Medicine, Guthrie Cortland Medical Center, Cortland, USA; 3 Internal Medicine, Wayne State University School of Medicine, Detroit, USA; 4 Internal Medicine, Dow University of Health Sciences, Karachi, PAK; 5 Internal Medicine, Carle Foundation Hospital, Urbana, USA; 6 Internal Medicine, The Wright Center for Graduate Medical Education, Scranton, USA; 7 Internal Medicine, Wayne State University, Detroit, USA; 8 Internal Medicine, Camden Clark Medical Center, Parkersburg, USA; 9 Cardiology, West Virginia University, Morgantown, USA; 10 Cardiology, Wayne State University Detroit Medical Center, Detroit, USA

**Keywords:** radiation prevention, radpad, cath lab, interventional cardiologists, radiation safety

## Abstract

One of the leading environmental hazards, ionizing radiation, is linked to several detrimental health consequences in the body. RADPAD (Worldwide Innovations & Technologies, Inc., Kansas City, Kansas) is a sterile, lead-free, lightweight, disposable radiation protection shield. We conducted a systematic review and meta-analysis to determine the effectiveness of RADPAD protection drapes in the cardiac catheterization lab and how they can aid interventional cardiologists in becoming subjected to less scatter radiation.

PubMed, Embase, and Google Scholar were searched for studies discussing the efficacy of RADPAD protection drapes in reducing radiation exposure to operators in the cardiac catheterization laboratory. A random-effects model was used to pool odds ratios (ORs) and 95% confidence intervals (CIs) for endpoints: primary operator exposure dose, dose area product (DAP), relative exposure, and screening time.

Our analysis included 892 patients from six studies. Compared to the No-RADPAD group, primary operator exposure dose (E) was significantly lower in the RADPAD group (OR: -0.9, 95% CI: -1.36 to -0.43, I^2 ^= 80.5%, p = 0.0001). DAP was comparable between both groups (OR: 0.008, 95% CI: -0.12 to -0.14, I^2 ^= 0%, p = 0.9066). There was no difference in the relative exposure (E/DAP) (OR: -0.47, 95% CI: -0.96 to 0.02, I^2 ^= 0%, p = 0.90) and screening time (OR: 0.13, 95% CI: 0.08 to 0.35, I^2 ^= 0%, p = 0.22) between the two groups.

The interventional cardiology laboratory is exposed to significantly less scatter radiation during procedures owing to the RADPAD protective drape. Consequently, all catheterization laboratories could be advised to employ RADPAD protective drapes.

## Introduction and background

Ionizing radiation is a major environmental toxin that has been associated with numerous adverse negative health effects in the body, ranging from skin lesions to various types of cancer. It has been shown that exposure to ionizing radiation can lead to tissue reactions (previously known as deterministic effects) or stochastic effects where the probability of occurrence of an outcome is dose-dependent [1]. The number of interventional cardiology procedures has been increasing significantly over the past decade, exposing interventional cardiologists to high cumulative doses of radiation [2]. Therefore, minimizing radiation exposure to the personnel in the interventional cardiology laboratory is a crucial issue. With the increasing popularity of minimally invasive procedures for cardiovascular diseases, catheter-based cardiac procedures like percutaneous coronary intervention (PCI) or transcatheter aortic valve replacement (TAVR) are being performed more frequently. Given the use of fluoroscopy guidance in such procedures, the inherent potential of increased radiation exposure must be considered. It has been shown that cardiologists and related paramedical staff accrue considerable cumulative radiation exposure throughout their careers with a non-negligible lifetime attributable risk of cancer [3,4]. As a result of this disproportionately increased ionizing radiation exposure, interventional cardiologists have a correspondingly increased prevalence of many health conditions, from localized issues like skin lesions and cataracts to chronic systemic problems, including hypertension, hypercholesterolemia, and cancer [5]. Given the demonstrated negative health impacts of catheterization laboratory-related radiation exposure, the current strategies and solutions for radiation protection must be re-evaluated.

Despite significant investment and research efforts by physicians and industry leaders to develop new devices and techniques for interventional cardiac procedures, there has been minimal progress in radiation protection technology over the past two decades [6]. Current strategies to reduce scatter radiation include placing the X-ray generator as far away as possible from the patient, lowering the frame rate of the image capture system, and using personal protective equipment, such as lead aprons, thyroid collars, glasses, lead caps, and arm shields. Various ceiling-suspended screens and table-mounted curtain shields also exist to minimize scatter radiation further. RADPAD (Worldwide Innovations & Technologies, Inc., Kansas City, Kansas) is a sterile, lead-free, lightweight, disposable radiation protection shield. The purpose of this meta-analysis is to provide an overview of the efficacy of RADPAD protection drapes in the cardiac catheterization laboratory and their role in reducing scatter radiation exposure to interventional cardiologists.

## Review

**Figure 1:**
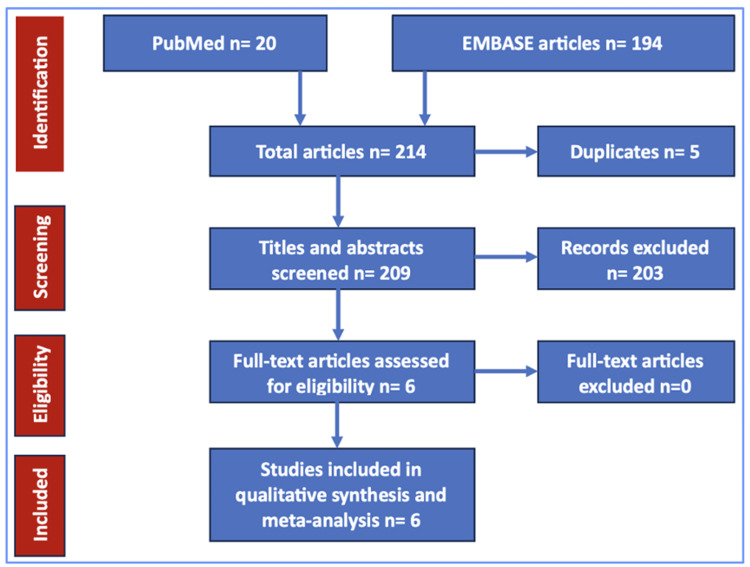
Flow of the search strategy for systematic review and meta-analysis using the Preferred Reporting Items for Systematic Reviews and Meta-Analyses.

This systematic review and meta-analysis followed the guidelines established by the Preferred Reporting Items for Systematic Reviews and Meta-Analyses (PRISMA) [7] and Assessing the Methodological Quality of Systematic Reviews-2 (AMSTAR-2) guidelines [8,9]. The checklists of these guidelines are shown in Figure 1, Supplemental S1, and Supplemental S2, respectively. A literature search was conducted on PubMed, Embase, and Google Scholar from inception till October 2023 using the following search terms: "radiation safety," "radiation protection," "cardiac catheterization," "percutaneous coronary intervention," and "interventional cardiologist." We used "OR" between two synonyms and "AND" between two different keywords. The studies were considered eligible for inclusion in our systematic review and meta-analysis if they satisfied the following criteria: (a) trials or observational studies discussing the efficacy of RADPAD protection drape in reducing radiation exposure to operators in the cardiac catheterization laboratory; (b) included adult male or female (≥18 years of age) participants; (c) reported at least one of the desired outcomes. Studies including case reports, clinical spotlights, and review articles were excluded during the screening.

Randomized clinical trials (RCTs), pilot trials, and retrospective and prospective studies that meet our inclusion guidelines were included. The papers were assessed independently by two authors (Khanal R. and Bahar A.), and full-text articles that passed screening were examined in a second screening phase for the assessment of relevant outcomes. To identify further papers to be included in the meta-analysis, we also performed backward snowballing, which involves looking through the reference sections of publications that may contain outcomes of interest. A third author (YS) independently reviewed the data screening after the initial screening.

Data and baseline characteristics were exported to Microsoft Excel (Microsoft Corporation, Redmond, WA), where they were organized into baseline characteristics, discrete variable outcomes in binary format, and continuous variable outcomes in a constant format. The baseline data of the included studies that were extracted had the author’s name, study design, the purpose of the study, type of intervention, results, and limitations. The characteristics of individual studies are shown in Table 1 [10-15]. The study outcomes include primary and secondary outcomes. The primary outcome was the direct operator exposure dose. In comparison, secondary outcomes were dose area product (DAP), relative exposure (E/DAP), and screening time.

**Table 1:** Characteristics of the studies included in the meta-analysis. PCI: percutaneous coronary intervention; CAST: cine adjusted screening time; AK: air kerma; DAP: dose area product; NOPAD: standard treatment; SHAMPAD: sham shield.

Author	Study design	Purpose of study	Intervention	Results	Limitation
Vlastra et al. [10]	Randomized clinical trial	Efficacy of RADPAD in the cardiac catheterization lab	RADPAD vs. NOPAD vs. SHAMPAD	20% reduction in radiation exposure to the primary operator using RADPAD	Exposure to head & eyes was not measured. Lack of patient dosimetry
Kherad et al. [15]	Randomized clinical trial	Efficacy of RADPAD shield in reducing the radiation dose experienced by the operator during diagnostic cardiac cath via femoral access	RADPAD vs. NO RADPAD	Overall, 59% reduction in radiation exposure to primary operators using RADPAD	Radiation levels were monitored only on the operator’s chest
Politi et al. [11]	Randomized clinical trial	The first randomized study in humans to test the efficacy of RADPAD designed to decrease the amount of radiation received by interventionists via the right radial approach	RADPAD vs. NO RADPAD	Significant reduction of radiation exposure to the operator using RADPAD	Radiation exposure was not evaluated in the left radial approach
Murphy et al. [12]	Randomized clinical trial	Assess the efficacy of RADPAD drapes in reducing radiation dose experienced by operators during prolonged, complex PCI procedures	RADPAD vs. NO RADPAD	Usage of RADPAD significantly reduced radiation exposure to primary operators during prolonged, complex PCI cases	The dosimeter was applied to the left arm only. The sham drape was not used
Shah et al. [14]	Randomized clinical trial	Radiation exposure reduction to operators with the use of RADPAD and measurement of radiation doses in different angiographic projections	RADPAD vs. NO RADPAD	39% reduction in relative operative exposure with the use of RADPAD	No sham drape was used for comparison. The dosimeter was not applied to secondary operators
MILD study [13]	Randomized clinical trial	Effect of using RADPAD on primary and secondary operators during coronary angiography and PCI	RADPAD vs. NO RADPAD	Dose exposure relative to CAST, AK, and DAP in primary and secondary operators was significantly lower (74%, 76%, and 79%) in the RADPAD group	Relatively small sample size and did not use sham drape

The pooled effect sizes were calculated using CRAN-R software, and the pooled odds risk was calculated using a meta-bin model along with the Mantel-Haenszel random-effects model. A probability value of p < 0.05 was considered statistically significant. A meta-cont module along with the inverse-variance method was used to calculate the pooled standard mean difference (SMD) with Hedges’ g for continuous outcomes with a probability value of p < 0.05 considered to be statistically significant. Higgins I2 was used to assess heterogeneity with values ≥ 75% indicating high heterogeneity and values of ≤ 50% corresponding to low to moderate heterogeneity [15].

Our thorough systematic search resulted in a total of 214 articles in the first phase. Following the removal of duplicates (n = 5), we screened the titles and abstracts of 209 articles in the first phase. Of these, 203 articles did not meet the inclusion criteria and were excluded. In the second phase of screening, six articles that reported the outcomes of interest for our analysis were included in the final analysis after screening comprehensively with a full-text review. All six studies were randomized controlled trials. Overall, we evaluated 892 patients (636 males and 256 females) undergoing cardiac catheterization. Patients were divided between RADPAD and No-RADPAD groups. Among the RADPAD group, 103 patients underwent femoral access, and 319 patients underwent radial access. Among the No-RADPAD group, 82 patients underwent femoral access, and 321 patients underwent radial access [9,11-14]. One study did not report access routes [10].

**Figure 2:**
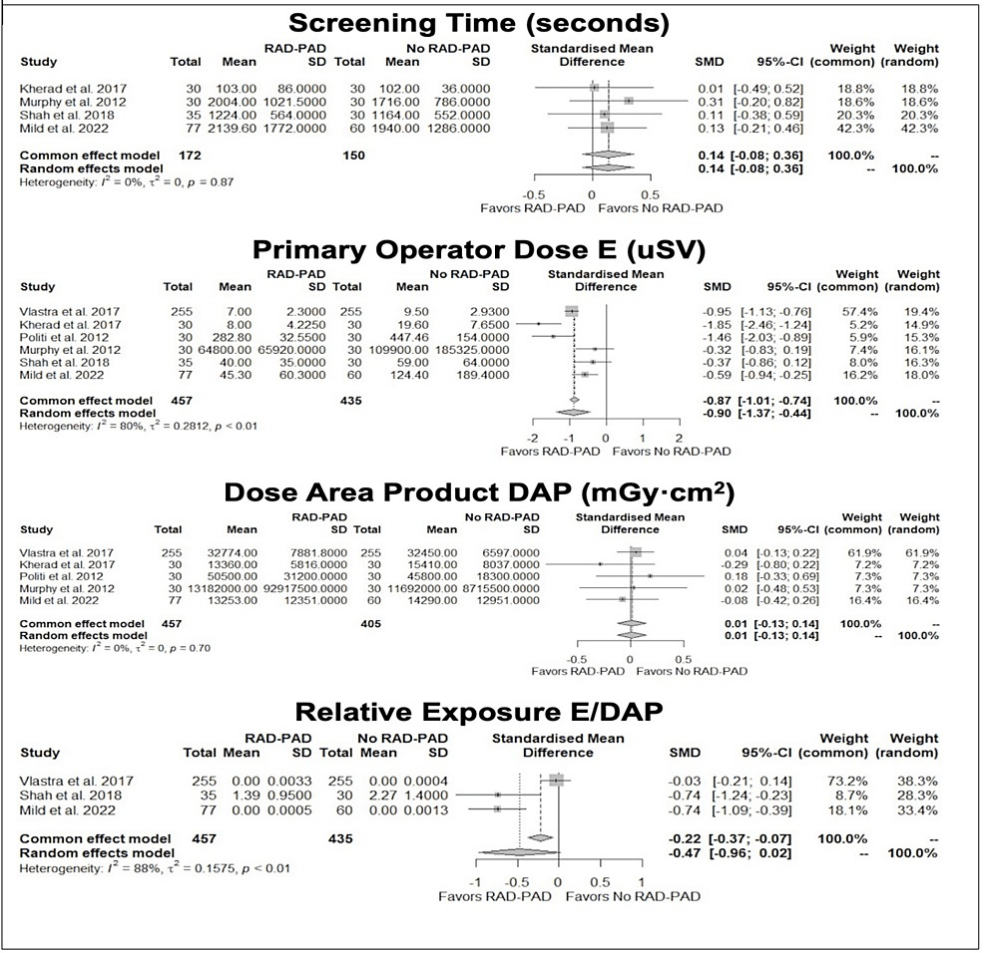
Comparison of screening time, primary operative dose, dose area product, and relative exposure between RADPAD and No-RADPAD groups. Comparison of RADPAD vs. No-RADPAD across six clinical trials: Kherad et al. [15], Murphy et al. [12], Shah et al. [14], MILD study [13], Vlastra et al. [10], and Politi et al. [11]. The figure illustrates the mean screening time (seconds), primary operator dose, dose area product, and relative exposure for procedures performed with and without RADPAD. Statistical significance is denoted by p < 0.05. RADPAD usage demonstrated a significant reduction in the primary operator’s exposure dose (OR: - 0.9, 95% CI: -1.36 to -0.43, I2 = 80.5%, p = 0.0001). There was no statistically significant difference between the two groups in terms of dose area product, screening time, and relative exposure (p > 0.05). DAP: dose area product; SMD: standard mean difference.

As shown in Figure 2, primary operator exposure dose (E) was significantly lower in the RADPAD group as compared to the No-RADPAD group in all six studies (OR: -0.9, 95% CI: -1.36 to -0.43, I2 = 80.5%, p = 0.0001). DAP was found to be comparable between both groups (OR: 0.008, 95% CI: -0.12 to -0.14, I2 = 0%, p = 0.9066). There was no difference in the relative exposure (E/DAP) (OR: -0.47, 95% CI: -0.96 to 0.02, I2 = 0%, p = 0.90) between the two groups. Screening time was comparatively shorter in the No-RADPAD group; however, it did not reach statistical significance (OR: 0.13, 95% CI: 0.08 to 0.35, I2 = 0%, p = 0.22) [9,11-14].

The revised Cochrane risk-of-bias 2 (RoB 2) tool for randomized trials was used for the assessment of bias in the included RCTs (Supplemental S4).

Given that all of the studied outcomes are reported by less than 10 studies, heterogeneity among the outcomes of the included studies is evident. As per the Cochrane Handbook for Systematic Reviews of Interventions, it is not possible to differentiate between true heterogeneity and findings merely by chance if the number of included studies is less than 10 [16]. Moreover, sampling error could explain the high percentage of variability.

Deterministic side effects usually occur once a certain threshold of ionizing radiation has been received. On the counterpart, stochastic effects are less predictable. They are a result of the damage to deoxyribonucleic acid (DNA) without leading to cell death. This damage to DNA can eventually lead to cell proliferation and subsequently neoplastic process. Although there is no specific or set threshold for stochastic effects, the risk is proportional to the dose [17]. In the interventional cardiovascular lab, the principal risks to be considered are stochastic risks of radiation-induced cataract formation and cancer as doses to the operator and staff do not typically approach thresholds of tissue reactions if the standard radiation protection tools are used [2].

Radiation-induced cataract is an important consideration in the realm of occupational and medical use of ionizing radiation. The exposures have historically been incurred in nuclear incidents, accidental exposures, and medical procedural settings [18]. Ionizing radiations include X-rays and gamma rays. These two types of high-energy radiation induce molecular and cellular changes in the lens. This leads to structural and functional changes in the epithelial and fiber cells of the lens culminating in cataract formation [18]. Radiation exposure also damages cellular DNA, which leads to a decrease in the production of cellular protective enzymes and altered intracellular protein concentrations. Radiation cataract primarily develops once a high dose threshold is exceeded [19]. However, several other studies have pointed toward cataract development at lower doses [20,21]. Healthcare workers involved in fluoroscopy-guided procedures are at the most significant risk of cataract formation. Among interventional cardiologists, radiation exposures in the cath lab particularly increase the risk of posterior subcapsular cataracts, although nuclear and cortical cataracts may also occur [21]. Regular eye exams are warranted to monitor changes in the lens. Preventive strategies include adopting measures to reduce unnecessary radiation exposure.

Radiation-induced cancer risk is well supported by the data from the Hiroshima and Nagasaki tumor registries [22]. Interventional cardiologists have a significant lifetime exposure to radiation ranging from 50 mSv to 200 mSv. This approximately correlates to 2500 to 10,000 chest X-rays [5]. With exposure to 100 mSv of radiation, there is an estimated 1% (0.3%-3%) incidence of radiation-induced cancer [5,22]. Andreassi et al. reported an adjusted odds ratio of 3.0 (0.6 to 13.7) of developing cancer in interventional cardiology staff compared to unexposed counterparts [5]. Among the different types of cancers associated with radiation, thyroid cancers, left-sided brain cancers, solid organ cancers, and leukemias have been described [9].

The risk of radiation-induced cancer depends on multiple variables in addition to radiation dose. The risk is higher in the younger population than the elderly and higher in females than males [22]. With the increase in the number and complexity of the cath-lab procedures requiring a longer duration of radiation, the risk of radiation-induced cancer is significant [17].

To the best of our knowledge, this paper is the first meta-analysis studying the efficacy of RADPAD in reducing the dose of radiation to the operator in the real world. Our results showed that the use of RADPAD protection drape significantly reduces scatter radiation exposure to primary and secondary operators in the cardiac catheterization laboratory during both simple and complex procedures.

Conventional lead aprons are heavy and uncomfortable and do not provide complete protection from scatter radiation especially to the eyes, brain, and arms [23]. RADPAD is a lead-free protection drape that absorbs scatter radiation from the patient by creating a shade zone for the catheterization lab personnel. It reduces scatter radiation to the operator, especially in areas that are not protected by usual protection devices. Operation of the drape from opening the package to positioning it on the patient takes less than 30 seconds [24]. Previous studies have demonstrated up to 72% reduction in the operator dose using a lead-free protection drape in a simulated cardiac cath lab operating through the radial route [25]. It has shown up to 86% reductions in scattered radiation dose to the operator in anthropomorphic phantom models [26]. The first randomized trial in humans was reported in 2012 by Politi et al. showing up to 34% reduction in radiation exposure to interventional cardiologists during diagnostic coronary angiography operating through the radial access with the utilization of RADPAD drape despite similar fluoroscopy time and total examination dose [11]. The study also suggested that the use of RADPAD is feasible given that the drape is simple to position and does not constitute a physical obstacle during the procedure. However, the fluoroscopy time was considerably short in the above study and the amount of radiation reduction obtainable could not be discerned if RADPAD protection drapes were used in more complex and lengthier procedures. The risk of exposure to scatter radiation increases exponentially in chronic total occlusion (CTO) cases given the requirement for relatively long cine runs for adequate visualization of the collateral circulation during PCI for CTO as well as multiple orthogonal imaging of the target artery during the wiring of CTOs. In a study performed by Murphy et.al, it was demonstrated that RADPAD significantly reduces the dose of scatter radiation to the primary operators during complex and prolonged cases such as multivessel PCI, rotational atherectomy, and CTOs [12]. However, the study was limited by the fact that radiation levels were measured only on the operator’s left arm and the radiation doses between the two groups could have possibly been different if it had been measured at different sites. Shah et al. also conducted a similar study of 65 randomly selected patients undergoing complex PCI with or without RADPAD drape, which demonstrated a 39% reduction in the relative operator exposure with the use of RADPAD [14]. Data from previous studies have shown that the highest attenuation of scatter radiation is obtained at the sites closest to the radiation source, such as arms and wrists, in comparison to dosimetry at chest and eye level [11,27]. Taking this into consideration, the study by Kherad et. al showed a 59% decrease in radiation exposure to primary operators during routine diagnostic coronary angiography performed via femoral access with the radiation levels measured at the operator’s chest [15]. The higher reduction rates in the radiation exposure to the primary operator in the above studies could be biased given the small study size and the fact that the dosimetry was performed at the left-arm level. However, the efficacy of RADPAD was further supported by the randomized control trial performed by Vlastra et. al in 2017, which included a relatively larger study population with the dosimetry performed at the chest level. In their study, a total of 766 coronary procedures were randomized to the use of RADPAD, standard treatment (NOPAD), or sham shield (SHAMPAD). The use of RADPAD was associated with a 20% reduction in relative operator exposure compared with that of NOPAD and a 44% relative exposure reduction compared with the use of SHAMPAD [10]. Studies have shown that left anterior oblique (LAO) oriented projections are the largest source of scatter radiation as compared to the rest of the views during coronary angioplasty [13,28]. The use of RADPAD could mitigate the risk of operator’s radiation exposure during procedures particularly requiring LAO views [13]. The secondary operator (SO) has a lower risk of scatter radiation exposure as compared to the primary operator (PO) due to the distance from the primary beam and source; however, the cumulative effects of radiation exposure to SO can have similar adverse effects as that of PO [29,30]. In the MILD study of 137 patients undergoing elective coronary angiography and PCI, the use of RADPAD was associated with a 64% reduction in radiation exposure to the PO and up to 79% reduction in the SO [13].

RADPAD has also been shown to reduce radiation exposure during other cardiovascular and non-cardiovascular procedures, such as cardiac resynchronization therapy, pacemaker and defibrillator placement, endovascular treatment of peripheral artery disease, and CT fluoroscopy-guided lung biopsy [29,31-33].

Limitations of the study include small sample sizes in four out of six studies and the wide range of cases that cause variability in fluoroscopy use. Also, it could be conceived that the use of the RADPAD drape made the operator more aware of radiation safety and that this may have made a bias during the dosimetry readings.

## Conclusions

The RADPAD protection drape significantly reduces scatter radiation exposure to the primary and secondary operators in the interventional cardiology laboratory during simple procedures as well as prolonged and complex procedures. It is a simple and convenient drape that could reduce the development of radiation-induced complications, including cataracts and cancer, in interventional cardiology personnel in the long term. Therefore, the use of RADPAD protection drapes should be recommended in all catheterization laboratories.

## Figures and Tables

**Figure 3:**
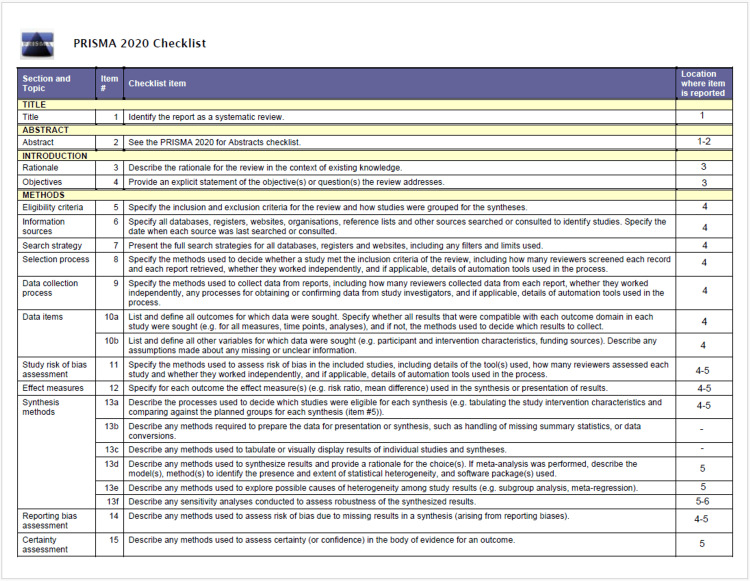
Preferred Reporting Items for Systematic Reviews and Meta-Analyses (PRISMA) checklist.

**Figure 4:**
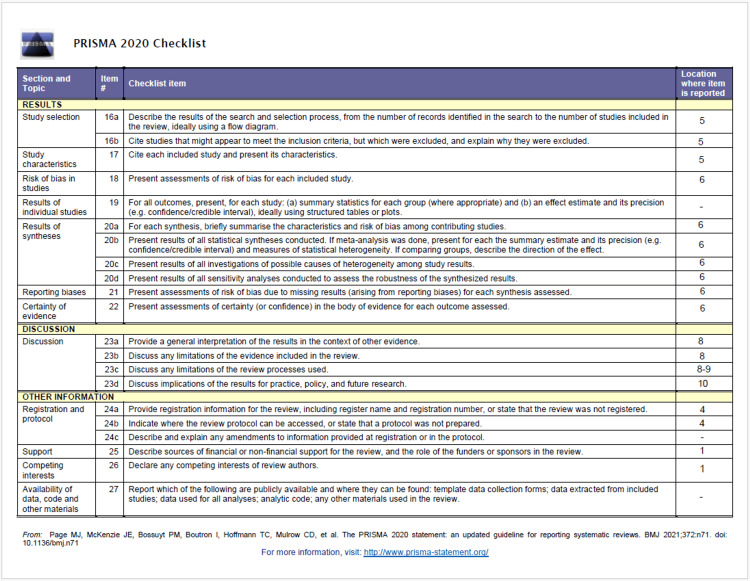
Preferred Reporting Items for Systematic Reviews and Meta-Analyses (PRISMA) checklist 2.

**Figure 5:**
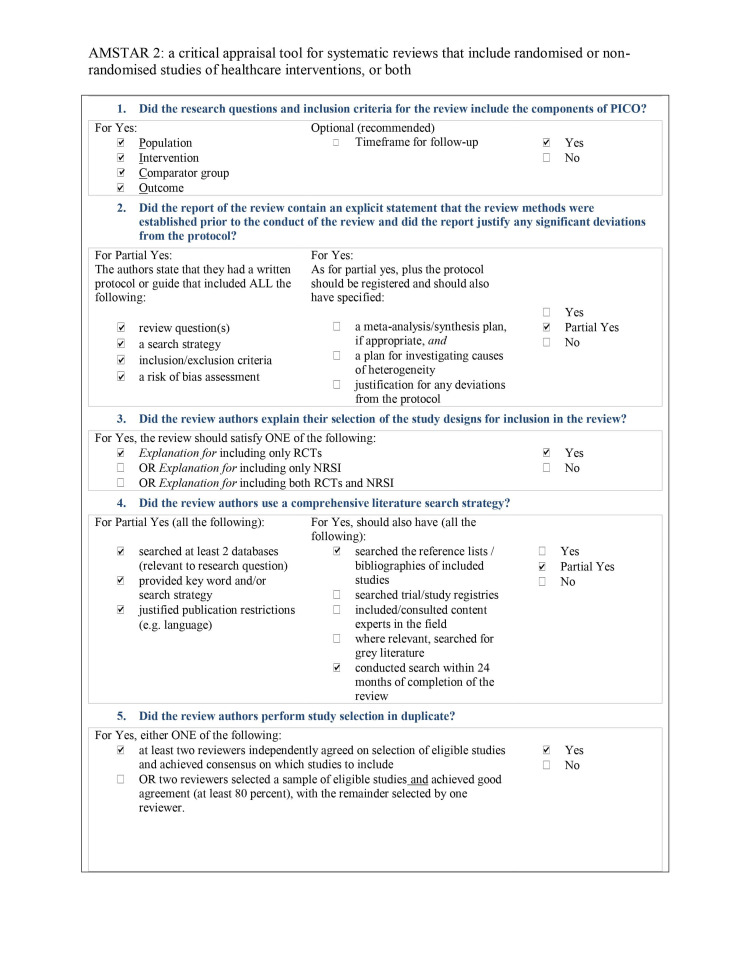
AMSTAR-2 (Assessing the Methodological Quality of Systematic Reviews-2) guidelines checklist.

**Figure 6:**
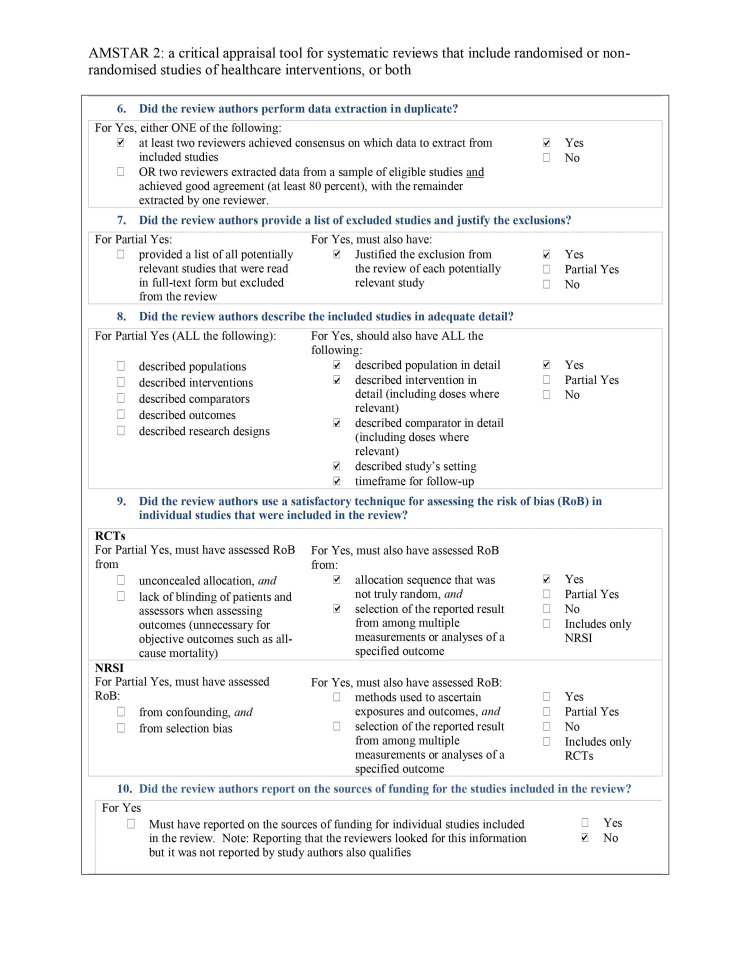
AMSTAR-2 (Assessing the Methodological Quality of Systematic Reviews-2) guidelines checklist.

**Figure 7:**
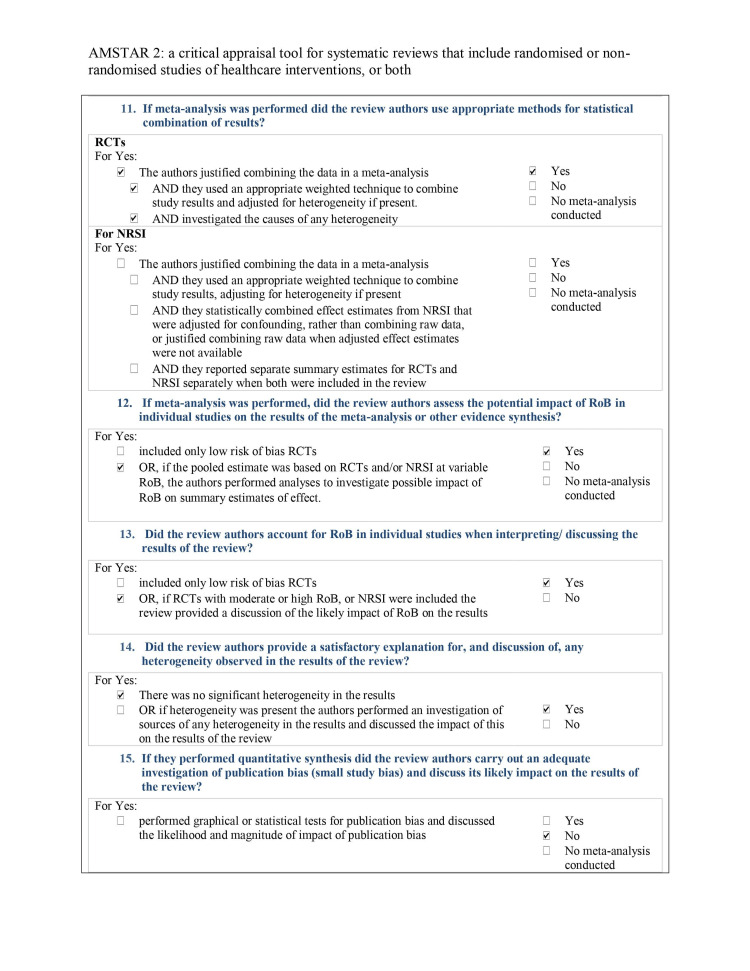
AMSTAR-2 (Assessing the Methodological Quality of Systematic Reviews-2) guidelines checklist.

**Figure 8:**
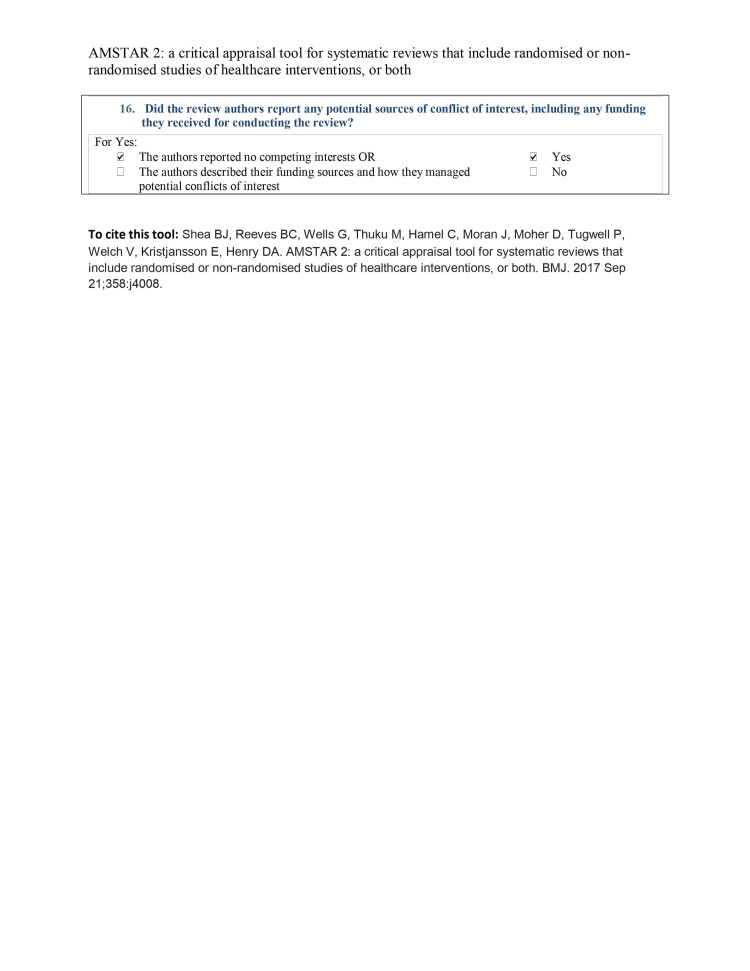
AMSTAR-2 (Assessing the Methodological Quality of Systematic Reviews-2) guidelines checklist.

**Figure 9:**
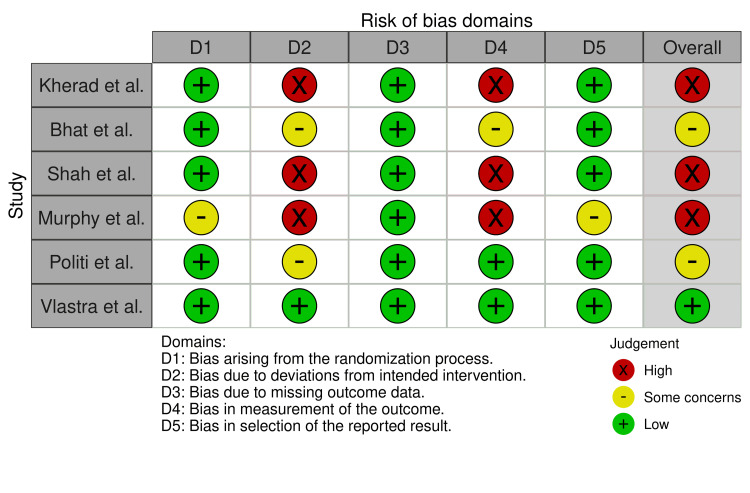
Cochrane risk-of-bias (ROB) tool assessment for included randomized controlled trials (RCTs). Assessing the risk of bias of the included randomized clinical trials. Overall, there is a low risk of bias in the study by Vlastra et al. [10], some concerns for bias in the studies by Politi et al. [11] and Bhat et al. [13], and a high risk of bias in the studies by Kherad et al. [15], Shah et al. [14], and Murphy et al. [12].

**Figure 10:**
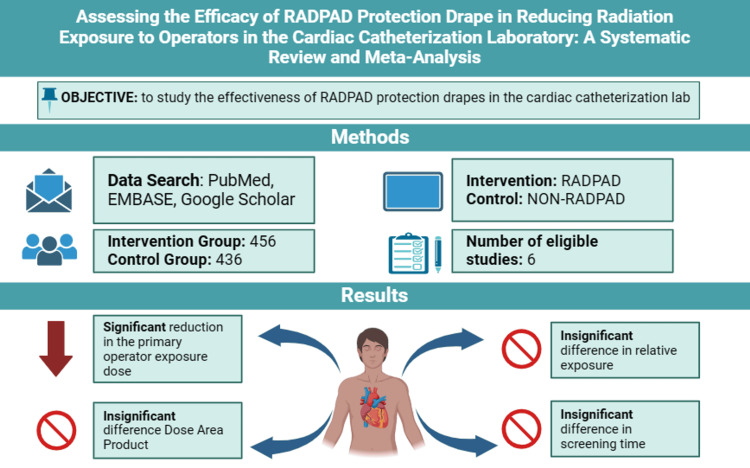
Graphical abstract.

## Appendices

Supplementary files index

1. Supplemental S1: Preferred Reporting Items for Systematic Reviews and Meta-Analyses (PRISMA) checklist

2. Supplemental S2: AMSTAR-2 (Assessing the Methodological Quality of Systematic Reviews-2) guidelines checklist

3. Supplemental S3: Research question, PICO (population, intervention, comparison, and outcomes), MeSH and keywords, and search strategy

4. Supplemental S4: Cochrane risk-of-bias (ROB) tool assessment for included randomized controlled trials (RCTs)

Supplemental S1

Supplemental S2

Supplemental S3

Research Question, PICO, MeSH Terms and Keywords, and Search Strategy

Research question: Assessing the efficacy of RADPAD protection drape in reducing radiation exposure to operators in the cardiac catheterization laboratory.

PICO: Population - operators in the catheterization laboratory. Intervention - RADPAD protection drape. Comparison - No RADPAD. Outcomes - (1) Primary outcomes: direct operator exposure dose. (2) Secondary outcomes: dose area product (DAP), relative exposure (E/DAP), and screening time.

Study type: Odds ratio to compare binary outcomes and standard mean difference to compare continuous outcomes meta-analyses.

MeSH terms & keywords: Radiation safety, radiation protection, radiation exposure, RADPAD, catheterization laboratory, interventional cardiologist, interventional cardiology, primary operator, and secondary operator.

Supplemental S4
